# A comparison of complementary and alternative medicine users and use across geographical areas: A national survey of 1,427 women

**DOI:** 10.1186/1472-6882-11-85

**Published:** 2011-10-07

**Authors:** Jon Adams, David Sibbritt, Alex Broom, Deborah Loxton, Marie Pirotta, John Humphreys, Chi-Wai Lui

**Affiliations:** 1 Faculty of Nursing Midwifery and Health, University of Technology Sydney, Sydney, New South Wales, Australia; 2 School of Medicine and Public Health, University of Newcastle, New South Wales, Australia; 3 School of Population Health, University of Queensland, Brisbane, Queensland, Australia; 4 School of Social Science, University of Queensland, Brisbane, Queensland, Australia; 5 Priority Research Centre for Gender, Health and Ageing, University of Newcastle, New South Wales, Australia; 6 School of Rural Health, Monash University, Bendigo, Victoria, Australia; 7 Department of General Practice, University of Melbourne, Victoria, Australia; 8 Network of Researchers in the Public Health of Complementary and Alternative Medicine (NORPHCAM), Faculty of Nursing, Midwifery and Health, University of Technology, Level 7 Building 10, 235-253 Jones St, Ultimo New South Wales, 2007, Australia

## Abstract

**Background:**

Evidence indicates that people who reside in non-urban areas have a higher use of complementary and alternative medicine (CAM) than people who reside in urban areas. However, there is sparse research on the reasons for such differences. This paper investigates the reasons for geographical differences in CAM use by comparing CAM users from four geographical areas (major cities, inner regional, outer region, rural/remote) across a range of health status, healthcare satisfaction, neighbourhood and community factors.

**Methods:**

A cross-sectional survey of 1,427 participants from the Australian Longitudinal Study on Women's Health (ALSWH) conducted in 2009.

**Results:**

The average total cost of consultations with CAM practitioners was $416 per annum and was highest for women in the major cities, declining with increasing distance from capital cities/remoteness (p < 0.001). The average total cost of self-prescribed CAM was $349 per annum, but this did not significantly differ across geographical areas. The increased use of CAM in rural and remote areas appears to be influenced by poorer access to conventional medical care (p < 0.05) and a greater sense of community (p < 0.05) amongst these rural and remote residents. In contrast to the findings of previous research this study found that health status was not associated with the differences in CAM use between urban and non-urban areas.

**Conclusion:**

It appears that a number of factors influence the different levels of CAM use across the urban/non-urban divide. Further research is needed to help tease out and understand these factors. Such research will help support health care policy and practice with regards to this topic.

## Background

Complementary and alternative medicine (CAM) is an umbrella term that refers to a broad group of healthcare systems, therapeutic practices and products (including acupuncture, chiropractic, naturopathy, herbal medicine and yoga) that are not traditionally associated with the conventional medical profession [1]. Large scale surveys suggest that CAM is increasing in popularity in North America, Australia and Europe in recent years [2-5] and such trends have generated much interest and debate among health professionals and policy-makers [6].

In recent years, an evidence-base that describes and evaluates CAM use has gradually emerged [7]. However, many of the available studies have focused on the use of CAM in metropolitan settings or amongst general populations [e.g. 3]. Although there is a growing interest in a geographical-perspective in CAM research focusing upon the dynamics of wellbeing, health practices and spatial locations [7-9], there is a lack of research examining CAM use in rural areas or the characteristics of CAM users across urban and non-urban residence [10]. This omission is perplexing as previous studies indicate that the prevalence of CAM use in rural and remote regions is higher than in urban areas [5,11-18].

One explanation put forward to account for differences in CAM use across the urban/non-urban divide has been poor access to and/or lack of satisfaction with conventional health services in non-urban regions [19-25]. However, there is a paucity of empirical research on how access difficulties affect decision about using CAM in rural areas [26]. Meanwhile, some work appears to highlight the critical role of a strong sense of neighbourhood and the strength of community network referral as well as localised beliefs regarding folk remedies in accounting for higher CAM consumption rates in non-urban areas [13,17,27-30]. In addition, there is evidence that women living in rural areas have a higher incidence of chronic illness and lower self-rated health status than women living in metropolitan areas [4,5,11,31-33].

Unfortunately, beyond these initial commentaries and early studies we still have little data to help explain differences in urban and non-urban CAM use. In response, this paper reports the findings of a survey comparing the characteristics of female CAM users living in urban and non-urban settings in Australia. The survey provides the first large-scale empirical comparison of a national sample of CAM users across rural and urban residence. Based on the study findings, the paper seeks to account for differences in urban/non-urban CAM use and address the gap in our understanding of CAM consumption across geographical areas.

## Methods

### *Sample*

This research was a sub-study of the Australian Longitudinal Survey of Women's Health (ALSWH). The ALSWH was designed to investigate multiple factors affecting the health and well being of women over a 20-year period. Relevant ethical approval was gained from the Human Ethics Committee at the University of Queensland and University of Newcastle, Australia. Women in three age groups were randomly selected from the national Medicare database [34] and invited by mail to participate. The focus of this study is women from the 1946-51 cohort aged 45-50 when the study started in 1996. At survey 1, 14,779 consented to participate and the respondents were shown to be broadly representative of the national population of women in the target age group, with some overrepresentation of women with higher levels of education and underrepresentation of women who have English as a second language [35]. For this sub-study, 2,120 women who had indicated in survey 5 (2007) that they consulted a CAM practitioner were mailed a questionnaire, of these women 1,800 (85%) returned completed questionnaires. From the 1,800 respondents, 1,427 women indicated that they still consult a CAM practitioner and it is these women that were included in the analyses for the current study.

### *Area of Residence*

The address of usual residence at each survey for each woman in the ALSWH has been geo-coded and allocated an ARIA+ remoteness score according to the Australian Standard Geographical Classification (ASGC) released in 2001 by the Australian Bureau of Statistics [36]. The ASGC classification categorises areas of residence as 'major cities', 'inner regional', 'outer regional', 'remote' and 'very remote' based on road distance from a locality to the closest service centre.

The participants of this study were categorised into 4 areas of residence: major cities (ARIA+ score: 0-0.20), inner regional (>0.20-2.40), outer regional (>2.40-5.92) and remote/very remote (>5.92). The categories of 'remote' and 'very remote' were combined in the analysis as the numbers of participants from these two areas were small. The sample consisted of 429 women from major cities, 437 women from inner regional areas, 470 women from outer regional areas, and 91 women from remote or very remote areas.

### *Measures of health status*

Women were asked how often they had sought help for a list of 24 symptoms (such as back pain, severe tiredness, depression, anxiety) in the previous twelve months. Women were also asked whether they had been diagnosed with any of 21 chronic medical conditions (such as diabetes, arthritis, heart disease, hypertension, breast cancer). The Short-Form 36 (SF-36) Quality of Life questionnaire was used to produce a measure of health status and quality of life [37]. Results of the SF-36 were reported in eight subscales [37]. The scores were standardised to a mean of 50 and standard deviation of 10, with higher scores representing better health [38].

### *Use of CAM*

The women were asked if they had consulted with a range of CAM practitioners or used a range of self-prescribed CAM for their own health in the previous 12 months. The list of CAM practitioners included: massage therapist, chiropractor, herbalist/naturopath, meditation/yoga therapist, acupuncturist, osteopath, reflexologist, spiritual health therapist, homeopath, traditional Chinese medicine therapist, aromatherapist, Ayurveda practitioner, music therapist. The list of self-prescribed CAM included: herbal medicines, vitamins/minerals, meditation/yoga, aromatherapy oils, Chinese medicine, prayer/spiritual healing. The women were also asked about the sources they used (e.g. family/relatives, friends/colleagues, internet) to obtain information regarding the CAM they consumed. The women were asked whether they inform their doctor or pharmacist before/after using CAM. They were also asked to estimate the total cost of their consultation(s) with CAM practitioner(s) and if applicable, the total cost of self-prescribed CAM used during the previous 12 months.

### *Neighbourhood safety and satisfaction*

Validated questions regarding neighbourhood safety and neighbourhood satisfaction come from the Healthy Communities Survey in Tasmania [39]. The questions asked about feelings concerning neighbours and neighbourhood, to assess the impact of neighbourhood connectedness on health and well being. Our questionnaire duplicated those of the Tasmanian study, with minor differences in wording of two items and omission of the response category of 'not applicable'. Neighbourhood connectedness was assessed using 13 items with higher scores reflecting greater satisfaction and greater safety [for more detail see 40].

### *Sense of community*

Questions regarding sense of community came from the Sense of Community Index 2 (SCI-2) [41] which is a modified version of the widely used Sense of Community Index in the social sciences [42]. The SCI-2 contains 25 items, where a higher total score reflects a greater sense of community. Previous application of SCI-2 has shown this measure to be reliable [41].

### *Rating of health care providers/services*

The women were asked to rate their level of satisfaction with various aspects of conventional health care providers (such as access to a female GP, hours when a GP is available, outcomes of medical care) and CAM providers (such as availability of CAM practitioners, ease of seeing CAM practitioner of one's choice). Each aspect was rated via a 5 point Likert scale, where 1 = excellent and 5 = poor. The women were also asked if they agreed with a series of statements regarding CAM (e.g. CAM is more natural than conventional medicine, CAM is a better preventive measure than conventional medicine). Each statement was rated via a 5-point Likert scale, where 1 = strongly agree and 5 = strongly disagree.

### *Statistical analyses*

Analysis of variance (ANOVA) tests and, when appropriate, the Bonferroni-Dunn *t*-test for multiple comparisons, were used to compare the area of residence variable against continuous variables. Chi-square tests and, when appropriate, pairwise post-hoc chi-square tests with Bonferroni adjustment, were used to compare the area of residence variable against categorical variables. All analyses were conducted using the statistical software SAS 9.1.

## Results

Of the 1,427 women who consulted a CAM practitioner in the previous 12 months, 43.6% consulted one CAM practitioner, 29.8% consulted two CAM practitioners, 13.5% consulted three CAM practitioners, and 13.1% consulted four or more different CAM practitioners. The most common CAM practitioners consulted were massage therapists (n = 912; 63.9%), chiropractors (n = 614; 43.0%), herbalists/naturopaths (n = 327; 22.9%), meditation/yoga therapists (n = 240; 16.8%), and acupuncturists (n = 215; 15.1%).

There were 1,292 (90.5%) women who used a self-prescribed CAM in the previous 12 months. Specifically, 32.7% used one form of self-prescribed CAM, 26.9% used two different forms of self-prescribed CAM, 16.5% used three different forms of self-prescribed CAM, and 14.4% used four or more different forms of self-prescribed CAM. The most common self-prescribed CAMs used were vitamins/minerals (n = 1,154; 80.9%), herbal medicines (n = 583; 40.9%), prayer/spiritual healing (n = 348; 24.4%), aromatherapy oils (n = 341; 23.9%), meditation/yoga (n = 334; 23.4%), Chinese medicine (n = 101; 7.1%).

Table 1 shows a comparison of area of residence against measures of CAM use, neighbourhood satisfaction and safety, sense of community, and health status. The average number of consultations with practitioners from different CAM modalities was highest in major cities and declined with increasing levels of rurality. However, this difference was not statistically significant. The average total cost of consultations with CAM practitioners was $416 per annum and was highest for women in major cities, declining with increasing levels of rurality (p < 0.001). The average number of different self-prescribed CAM used by the women did not differ between areas of residence and nor did the average total cost of self-prescribed CAM ($349 per annum).

**Table 1:** Comparisons of area of residence against mean (SD) measures of CAM use, Neighbourhood satisfaction and safety, sense of community, and health status

Characteristic	Major Cities	Inner Regional	Outer Regional	Remote/Very Remote	p-value
	**(n = 429)**	**(n = 437)**	**(n = 470)**	**(n = 91)**	

**Number of different CAM modalities**	2.2 (1.5)	2.1 (1.3)	2.0 (1.3)	1.9 (1.1)	0.3260

					

**Total cost of consultations **^A B C^	488.1 (441.2)	389.4 (384.1)	386.9 (364.0)	356.3 (333.3)	<0.0001

					

**Number of different self- prescribed CAMs**	2.1 (1.4)	2.0 (1.4)	1.9 (1.3)	2.1 (1.3)	0.4503

					

**Total cost of self-prescribed CAMs**	384.2 (393.8)	338.3 (336.2)	333.6 (336.2)	320.4 (305.9)	0.1360

					

**Neighbourhood safety **^A B C^	4.6 (1.4)	4.4 (1.4)	4.4 (1.6)	4.2 (1.8)	0.0036

					

**Neighbourhood satisfaction**	15.8 (4.3)	15.3 (4.1)	15.1 (4.4)	14.8 (4.7)	0.0557

					

**Total Sense of Community Index **^A B C^	25.5 (13.1)	30.0 (13.0)	31.8 (14.1)	34.0 (15.3)	<0.0001

					

**Number of diagnoses**	1.6 (1.5)	1.6 (1.5)	1.6 (1.6)	1.5 (1.5)	0.8726

					

**Number of symptoms**	5.9 (4.1)	6.1 (4.1)	6.0 (4.0)	5.8 (3.8)	0.1536

^A ^statistically significant difference between major cities and inner regional (p < 0.05)

^B ^statistically significant difference between major cities and outer regional (p < 0.05)

^C ^statistically significant difference between major cities and remote/very remote (p < 0.05)

^D ^statistically significant difference between inner regional and remote/very remote (p < 0.05)

There were no statistically significant differences between areas of residence and the average number of symptoms or diagnoses. In terms of SF-36 quality of life measures (data not shown), only one of the eight dimensions, physical functioning, was statistically different across the areas of residence (p < 0.001). That is, women in major cities had better physical functioning than women in rural areas, particularly women from outer regional areas.

For these CAM users, there was a statistically significant difference in the mean values for neighbourhood safety (p < 0.005) with women from major cities feeling safer than women from inner regional areas, outer regional areas and remote or very remote areas. There were also statistically significant differences between area of residence and sense of community (p < 0.0001). Specifically, in comparison to women in inner regional areas, outer regional areas and remote or very remote areas, women in major cities had a poorer total sense of community (p < 0.05). There was no significant difference between areas of residence and neighbourhood satisfaction.

Comparisons of area of residence against information sources for CAM, information sharing about CAM use, attitudes towards CAM, and satisfaction with health care are provided in Table 2. There is a statistically significant association between the use of family or relatives as an information source for CAM and area of residence (p < 0.005). Specifically, a greater percentage of women from remote or very remote areas use family or relatives as an information source for CAM compared to women from outer regional areas (p < 0.05). Similarly, a greater percentage of women from major cities use family or relatives as an information source for CAM compared to women from outer regional areas (p < 0.05).

**Table 2:** Comparisons of area of residence against information sources for alternative medicine, information sharing about alternative medicine use, and satisfaction with health care

Characteristic	Major Cities	Inner Regional	Outer Regional	Remote/Very Remote	p-value
	(n = 429)	(n = 437)	(n = 470)	(n = 91)	
	**%**	**%**	**%**	**%**	
**Information source for**					
**alternative medicine**					
Family/relatives (% yes) ^B F^	39	34	30	45	0.0044
Partner (% yes)	10	10	12	16	0.2131
Friends/colleagues (% yes) ^A B^	47	39	37	45	0.0207
Internet (% yes) ^A^	11	5	8	8	0.0255
Book or magazine (% yes)	29	27	30	33	0.5665
Mass media (% yes) ^A E F^	14	9	10	20	0.0071
Doctor (% yes) ^A B^	33	26	23	25	0.0047
Pharmacist (% yes)	13	14	14	13	0.9603
Allied health worker (% yes)	5	3	5	7	0.4137
CAM health practitioner (% yes)	25	29	25	20	0.1905
					

**Satisfaction with health care**					
**(% good/very good/excellent)**					
Access to specialists ^A B C D E^	91	83	69	60	<0.0001
Access to a GP who bulk bills^A C^	57	46	51	44	0.0074
Access to a female GP ^A B C^	76	66	62	59	<0.0001
Hours when GP is available ^B C^	72	66	60	58	0.0023
Number of GPs you have to choose from ^A B C D E F^	79	67	59	42	<0.0001
Ease of seeing the GP of your choice ^A B C D^	70	60	52	49	<0.0001
How long you wait to get a GP appointment ^A B C^	69	53	49	47	<0.0001
Quality of care provided by your GP	91	92	89	86	0.1478
Amount of time for a GP consultation	80	77	74	73	0.1209
Amount of information sharing by GP^B C D E^	83	83	76	69	0.0005
The outcomes of your medical care	87	85	82	80	0.1553
The technical skills of your GP	92	94	88	90	0.0506
Availability of CAM practitioners in your community ^A B C D E F^	79	71	56	38	<0.0001
Ease of seeing the CAM practitioner of choice ^B C D E F^	75	69	53	42	<0.0001
Access to information about CAM ^B C D E^	70	64	51	44	<0.0001

^A ^statistically significant difference between major cities and inner regional (p < 0.05)

^B ^statistically significant difference between major cities and outer regional (p < 0.05)

^C ^statistically significant difference between major cities and remote/very remote (p < 0.05)

^D ^statistically significant difference between inner regional and outer regional (p < 0.05)

^E ^statistically significant difference between inner regional and remote/very remote (p < 0.05)

^F ^statistically significant difference between outer regional and remote/very remote (p < 0.05)

As shown in Table 2, obtaining information about CAM from family and relatives or from friends and colleagues was more common among those women living in cities and remote areas than women living in regional areas (p < 0.05). Obtaining CAM information from the internet or from a doctor was most common among women living in major cities (p < 0.05). Obtaining information from a mass media source was most common among women living in remote areas (p < 0.05). There were no significant differences by area for obtaining information from partners, books or magazines, pharmacists, allied or CAM practitioners.

Women from remote or very remote areas were the least likely to report satisfaction with access to services and to some aspects of the quality of those services (i.e. hours of GP availability, number of GPs available, choice of GP & delay in seeing a GP, amount of information sharing with GP) (Table 2). Overall, satisfaction with access to medical practitioners and with quality of service was highest among women from major cities. There were no differences by geographical area in regards to satisfaction with the quality of GP care provided, the amount of time spent in consultation with a GP, GP technical skills, or the outcomes of medical care.

Moving across the geographical spectrum from urban to remote residence, the level of availability, access to CAM practitioners and information about CAM all appear to decrease (Table 2). Women from major cities were significantly more likely to report availability of CAM services, ease of seeing a CAM practitioner of their choice and access to information about CAM compared to women from all other areas. Less than half of the women living in remote areas and around half of those living in regional areas reported satisfactory access to CAM services, compared with 70-75% of those living in major cities who reported such satisfactory access.

## Discussion

This is the first large-scale study to compare a national sample of CAM users across urban and non-urban residence in Australia. Although this study focuses specifically on mid-age women, previous research has identified this group as being high CAM users. Although the interpretation of results is limited by the fact that the health care utilisation data is self-reported by the participants and open to recall bias or errors, such a limitation is by far outstripped by the insight gained through collecting and analysing a large, nationally-representative sample of CAM users. Specifically, the findings that a substantial percentage of participants consulted with more than one type of CAM practitioner (56%) and used more than one form of self-prescribed CAM (67%) are consistent with the results of previous studies on the general CAM population [2,3]. These findings confirm that CAM consumption has become a mainstream health care activity in Australia as in other Western countries [7].

As there is a lack of empirical testing of accounts for higher CAM use in rural areas, this study provides a unique opportunity to create an evidence base for assessing the adequacy of proposed explanations (the higher incidence of chronic illness and lower self-rated health status among women living in rural areas, poor access/low satisfaction with conventional health services in non-urban regions, and a stronger sense of neighbourhood and the strength of community network referral in remote settings) to account for differences in prevalence of CAM use between urban and non-urban regions.

In contrast to previous research findings illustrating an association between poor health and CAM use [4,5,11,31], this study found no significant differences in the health status of CAM users across geographical regions. This result casts some doubt on the role of health status as an influence on women's CAM use across the urban/non-urban divide. However, the association between health status and geographical CAM use is a complex issue as there is evidence that the association between poor health and increased CAM use may vary depending on the frequency and length of CAM consumption [43]. It is recommended that further studies be conducted to explore this issue in more depth.

The study findings provide evidence of a link between access to conventional health care and the adoption of CAM, with poor access to such health services predicting higher CAM use in non-urban regions. In particular, the study identifies significant differences between urban and non-urban CAM users in terms of their satisfaction with access to specialists, hospitals, after-hours care, female GPs or bulk billing GPs. The results also suggest a decrease in satisfaction with access to all these services and practitioner types with increasing levels of remoteness. Together these findings provide support to the claim that a lack of access to and/or patient dissatisfaction with conventional health practitioners play an important role in accounting for the higher use of CAM in rural or remote areas [19-23]. However, it is important to note that the level of satisfaction with access to CAM practitioners was also poorer in non-urban regions. Cleary, further research is required to examine access and satisfaction with health care across regions. If a link does exist between CAM use and lack of access to affordable, quality conventional health care, then CAM may constitute a health care safety net for non-urban patients that have hitherto escaped the focus of providers and policymakers. An understanding of CAM use in these settings may provide valuable insight for planning and improving the coordination of care and collaborations between existing resources in non-urban health care systems [10,24,25].

The results of this survey also shed further light on the role of a sense of neighbourhood and community network in shaping CAM consumption trends across the urban/non-urban divide. The findings indicate that although the participating CAM users in major cities feel safer in their neighbourhood, they also have a poorer sense of community than their counterparts living in regional and remote areas. Having a stronger sense of neighbourhood implies that people living in non-urban regions have closer personal relationships and are more likely to share experiences and provide information among each other [44-46]. This falls in line with our finding that women from remote and very remote areas rely heavily on family, relatives and the mass media as sources of CAM information and provides further support to the claim that a stronger sense of neighbourhood and social connectedness helps facilitate or proliferate the use of CAM in rural regions. Our results confirm previous research findings suggesting that geographical factors influence how people acquire and receive information about CAM [47,48]. Further in-depth qualitative research is needed to examine the nature of CAM information seeking and sharing as well as community network referral systems in rural locations [13,17,27-29,49,50].

The study found no significant difference in the average number of CAM practitioner consultations or any difference in the consumption of self-prescribed CAM across residence (urban, rural and remote). However, the results reveal that the amount users spend on CAM consultations does decline with increasing levels of rurality. One possible explanation for this difference is the variations in consultation fees across urban/non-urban regions (with practitioners charging less in non-urban areas). Ultimately, this expense issue requires further research along the lines of economic analysis of CAM practitioner fees/patterns of practice, and qualitative research examining the intricacies of women's experiences, perceptions and motivations for CAM consumption.

## Conclusions

This study is the first large-scale research undertaken to compare CAM users across urban and non-urban residence in Australia. In addition to improving our understanding of CAM consumption across geographical areas, the study findings also help identify the factors that influence the different levels of CAM use across the urban/non-urban divide.

When considered as a whole, the study findings suggest a need to investigate further possible influences beyond user attributes or values to account for differences in the use of CAM across urban/non-urban residence. This includes an examination of the influences of informal community networks, local cultural beliefs and closer community ties on the use and knowledge of CAM. Further research is needed to help tease out and understand these aspects of CAM consumption and the findings from such research will help support health care policy and practice with regards to this topic.

## Competing interests

The authors declare that they have no competing interests.

## Authors' contributions

All authors devised the study and helped to conceptualize ideas, interpret findings, manuscript writing and reviewing drafts of the article. DS undertook data analysis and JA, DS, AB, DL, MP, JH, CL contributed to the interpretation of the data. All authors read and approved the final manuscript.

## Pre-publication history

The pre-publication history for this paper can be accessed here:

http://www.biomedcentral.com/1472-6882/11/85/prepub
